# Ecosystem engineering by digging mammals: effects on soil fertility and condition in Tasmanian temperate woodland

**DOI:** 10.1098/rsos.180621

**Published:** 2019-01-16

**Authors:** G. T. O. Davies, J. B. Kirkpatrick, E. Z. Cameron, S. Carver, C. N. Johnson

**Affiliations:** 1 School of Natural Sciences, University of Tasmania, Private Bag 55, Hobart, Tasmania 7001, Australia; 2 Australian Research Council Centre of Excellence for Australian Biodiversity and Heritage, University of Tasmania, Private Bag 55, Hobart, Tasmania 7001, Australia; 3 Discipline of Geography and Spatial Sciences, University of Tasmania, Private Bag 78, Hobart, Tasmania 7001, Australia; 4 School of Biological Sciences, University of Canterbury, Private Bag 4800, Christchurch 8140, New Zealand

**Keywords:** ecosystem services, marsupial, monotreme, soil disturbance, ecological restoration

## Abstract

Many small- and medium-sized mammals dig for their food. This activity potentially affects soil condition and fertility. Digging is well developed especially in Australian mammals, many of which have recently become rare or extinct. We measured the effects of digging by mammals on soil in a Tasmanian temperate dry sclerophyll forest with an intact mammal community. The density of diggings was 5812 ha^−1^, affecting 11% of the forest floor. Diggings were created at a rate of around 3113 diggings ha^−1^ yr^−1^, disturbing 6.5% of the forest floor and displacing 7.1 m^3^ ha^−1^ of soil annually. Most diggings were made by eastern bettongs (*Bettongia gaimardi)* and short-beaked echidnas (*Tachyglossus aculeatus*). Many (approx. 30%) fresh diggings consisted of re-excavations of old diggings. Novel diggings displaced 5 m^3^ ha yr^−1^ of soil. Diggings acted as traps for organic matter and sites for the formation of new soil, which had higher fertility and moisture content and lower hardness than undisturbed topsoil. These effects on soil fertility and structure were strongest in habitats with dry and poor soil. Creation of fine-scaled heterogeneity by mammals, and amelioration of dry and infertile soil, is a valuable ecosystem service that could be restored by reintroduction of digging mammals to habitats from which they have declined or gone extinct.

## 1. Introduction

Ecosystem engineers are species that have strong effects on ecological communities by causing physical changes to the environment, which create, modify or maintain habitat for other species [1]. Bioturbation—the physical displacement of soil or sediment by organisms—is an important form of ecosystem engineering because of its effects on small-scale topography and the development of soil [2]. Digging by vertebrates can be an important mechanism of bioturbation and may have effects that include acceleration of material flows in ecosystems and creation of regeneration niches for plants [2,3]. Most research on the effects of digging by mammals has focused on burrowing species [3]. Burrowing has strong and semi-permanent effects on microtopography [4], on availability and distribution of nutrients [5,6], and consequently on biotic communities [5–7]. Burrowing can cause turnover of massive amounts of soil at landscape scales. For example, pocket gophers (*Geomyidae* spp.) in North America excavate around 18 m^3^ ha^−1^ yr^−1^ of soil [8,9].

Many mammals forage by digging, creating excavations that are smaller and more ephemeral than burrows, with effects that are likely to be subtle compared with burrowing. Nonetheless, diggings can occur at high density [10–13] and turn over large volumes of soil [3]. For example, in Switzerland, grubbing by wild boar (*Sus scrofa)* may disturb 27–54% of the forest floor [14]; American badgers (*Taxidea taxus)* displace soil at 5.1 t ha^−1^ yr^−1^ [12]; Cape porcupines *Hystrix africaeaustralis* make up to 3463 diggings ha^−1^ yr^−1^ and displace 1.6 m^3^ ha^−1^ yr^−1^ [15]. These activities could have large cumulative effects on ecosystems.

In Australia, many medium-sized mammals forage by digging [16]. These mammals include echidnas (*Tachyglossus aculeatus*) and several species of rat-kangaroos (Potoroidae) and bandicoots (Peramelidae), which dig for patchily distributed resources such as subterranean invertebrates or fungi. Foraging by these animals creates many intermediate-sized pits, which gradually refill with soil and leaf litter. This cycle of pit excavation and soil re-formation may change soil structure, microtopography, and the structure and biomass of the litter layer; it generates fine-scaled habitat heterogeneity [16]. However, on mainland Australia, it is difficult to evaluate the magnitude of these effects, because many of the excavating mammals have become rare or extinct due to impacts of introduced predators, especially the red fox *Vulpes vulpes* [16,17]. Invasive predators have had their greatest impacts on Australian mammals in the so-called critical weight range, between about 35 and 5500 g [18,19]; this body-mass range includes the majority of species that dig for their food.

Tasmania provides an opportunity to establish the ecological effects of digging mammals because, in the absence of the red fox, the community of medium-sized mammals is largely intact, and most species remain common throughout their original ranges. The mammalian fauna of Tasmania includes five species that weigh between 1 and 5 kg and feed almost entirely by digging: the eastern bettong (*Bettongia gaimardi*) and long-nosed potoroo (*Potorous tridactylus*), which feed mainly on subterranean fungi [20,21], the eastern barred bandicoot (*Perameles gunnii*) and southern brown bandicoot (*Isoodon obesulus*), which dig for invertebrates and fungi [20], and the short-beaked echidna, which digs for invertebrates [22]. These species typically feed by excavating discrete foraging pits that may be 15 cm (or more) deep while creating adjacent mounds of soil thrown out of the pits (authors' observations from this study).

We aimed to determine the magnitude and pattern of impacts on soil of an intact assemblage of native digging mammals in a temperate dry sclerophyll forest ecosystem in southeast Tasmania. Our study area included a range of forest types on a soil-fertility gradient, allowing us to test whether the effects of digging mammals on soil differed according to soil characteristics. We measured the densities of diggings and their rate of production to estimate the total physical effect of soil displacement and disturbance of the soil surface. We then compared the composition and structure of soil that formed as a result of the infilling of pits with soil from matched undisturbed sites and from spoil heaps created by mammals as the pits were excavated.

## 2. Methods

### 2.1. Study site

We worked in the Gravelly Ridge Conservation Area (2300 ha) near the town of Colebrook in southeast Tasmania (42°33′ S; 147°30′ E). The topography of the area is characterized by parallel ridges divided by often steep-sided gullies, over an elevation range from 200 to 400 m. The eastern part of the area is underlain by Permian silty sandstone (with thin sandy loam and yellowish clayey soils), with some quartz siltstone (with thin, fine, grey, clayey soils rich in gravel) and occasional dolerite deposits and pebble and mud conglomerates. The western part is underlain by Triassic quartz sandstone (thin sandy loam and yellow/orange clayey soils), shale (brown, muddy soils) with occasional siltstone, mudstone and sandstone deposits. Average rainfall is 630 mm, distributed evenly throughout the year. Dominant tree species are *Eucalyptus tenuiramis*, *E. obliqua* and *E. amygdalina*, with a mid-storey typically including *Acacia dealbata, A. melanoxylon, A. mucronata* and *Exocarpos cupressiformis*. The understorey is generally sparse and open, consisting mainly of small scleromorphic shrubs and grasses.

We distinguished four habitat types using a habitat classification developed for the larger region by the Tasmanian Forest Practices Authority [23], as follows:

(1) *Eucalyptus amygdalina* grassy forest: canopy dominated by *E. amygdalina* with understorey dominated by the tussock-forming *Lomandra longifolia* and grasses;
(2) *Eucalyptus amygdalina* shrubby forest: canopy dominated by *E. amygdalina* with understorey of tall shrubs and sedges;
(3) *Eucalyptus obliqua* shrubby forest: canopy dominated by *E. obliqua* with some *E. amygdalina*, and understorey dominated by tall wet and dry sclerophyll shrubs with a mid-storey of *Exocarpos cupressiformis* and *Acacia* species;
(4) *Eucalyptus tenuiramis* heathy forest: canopy dominated by *E. tenuiramis* with some *E. obliqua* and an understorey of low shrubs with patches of bracken (*Pteridium esculentum*) and rare taller shrubs.

The two *E. amygdalina* habitat types were rare in the study area, so we combined them into a single category of *E. amygdalina* forest. This forest type occurred on the more fertile soils of the area (see Results). Both *E. obliqua* forest and *E. tenuiramis* forest occurred on less fertile soils, and otherwise represented a gradient from wetter (*E. obliqua*) to drier *(E. tenuiramis*) habitats. Medium-sized mammals occurring in the area included the short-beaked echidna, southern brown bandicoot, eastern barred bandicoot, eastern bettong and long-nosed potoroo. Estimating population densities for these species was beyond the scope of this study, but a study of the eastern bettong population of the area in 2016 and 2017 obtained a density estimate of 11.5 individuals per km^2^ (R. Gardiner, K. Proft & S. Comte 2018, personal communication).

### 2.2. Data collection

We located sample plots using a map grid and a randomly generated set of coordinates. Twenty sample plots were in the western section of the Conservation Area, spread over 200 ha, and 20 in the eastern section spread over 600 ha. The number of plots in the three habitat types reflected the area of each: seven plots in *E. amygdalina* forest, 19 in *E. obliqua* forest and 14 in *E. tenuiramus* forest. The first 20 plots were 20 × 5 m. Two further plots were 10 × 5 m and the remaining 18 were 5 × 5 m. Resampling within large plots showed that plot area did not affect estimates of digging density (*p* = 0.125, *t*_19_ = 1.605); therefore, all plots were treated in the same way in the analysis. In each plot, all diggings were marked using a short wire peg to which a length of flagging tape was tied, and identified by a number code written on the tape, and locations of diggings within the plot were mapped. Each digging was assigned a value for relative age on a ten-point scale: zero value was given for freshly dug diggings (with no in-filling of the pit by organic matter, and the spoil heap still loose and clear of litter) and nine was given for diggings that could no longer be distinguished from the surrounding soil surface but had been identified and marked earlier in the study. Precise identification, mapping and age-indexing of diggings allowed us to identify the appearance of new diggings and to distinguish cases of re-excavation of old diggings when plots were re-surveyed. The species responsible for excavating the digging was identified where possible by careful examination of digging direction and style, associated tracks and signs [24], and by comparison to pits where species identity had been confirmed by remote cameras.

Each digging consisted of an excavated pit and associated spoil heap. We measured the long axis of each digging and the short axis perpendicular to it at its widest point and used these dimensions to calculate the surface area of an imaginary rectangle surrounding the disturbance. The percentage of this area that was disturbed was then estimated, to derive a value for the surface area of the disturbance. The dimensions of the pit and spoil heap were measured individually using the same method. Pit depth was also measured, and minimum pit volume was calculated by assuming that the space was an inverted elliptical cone, such that

$$\text{volume}=\pi \frac{0.5\;\text{length}*0.5\;\text{width}*\text{depth}}{3}.$$

Measurements were repeated four times over one year (once every three months) in each study site; October 2012–November 2013 in the west and February 2013–March 2014 in the east.

Soil samples were collected just outside (within 5 m) all plots so as not to disturb other sampling. Samples were taken from (i) soil formed in the pit of older diggings in which litter trapped by the pit had at least partially decomposed into soil, (ii) the freshly excavated spoil heaps of newer diggings and (iii) undisturbed topsoil. Each soil sample (i, ii and iii) from each plot comprised a subsample of 30 homogenized soil samples. The maximum diameter for pits selected for soil sampling was 15–20 cm. The samples were taken using a 2.5 inch soil corer. Triplets of samples (i, ii and iii) were taken within a 1 m^2^ area. The chemical characteristics (table 1) of each of 120 homogenized samples (three sample types from 40 plots) were determined by a commercial laboratory (CSBP Laboratory: https://www.csbp-fertilisers.com.au/agronomy/lab) using standard methods. Soil penetration resistance was measured using a penetrometer top to determine penetrometer force (Kgf cm^3^). Thirty random measurements within each plot of neighbouring (within 2 m) triplets of (i) soil in pits, (ii) spoil heaps and (iii) undisturbed topsoil, were made. The same process was repeated using a moisture probe to estimate soil moisture (% by weight). All measurements of soil moisture were taken within a single 4 day period and the number of days elapsed since last rainfall was recorded for each measurement day.

**Table 1.** Correlation coefficients describing relationships between original soil variables and scores for the first three principal components describing variation in the composition of soil samples (75% of variance cumulatively explained); strong correlations (*r* > 0.7) are shown in italics*.*

measurement	PC 1	PC 2	PC 3
pH	0.15	*0.80*	−0.43
conductivity	*0.83*	−0.10	0.04
ammonium nitrogen	0.27	0.16	*0.81*
nitrate nitrogen	0.42	0.54	−0.29
phosphorus	*0.78*	0.28	0.34
potassium	*0.77*	−0.25	−0.42
sulfur	*0.82*	−0.38	0.18
organic carbon %	*0.77*	−0.20	0.14
DTPA copper	*0.73*	0.46	−0.04
DTPA iron	0.61	−0.66	0.05
DTPA manganese	0.31	*0.77*	0.27
DTPA zinc	*0.85*	0.14	0.30
aluminium	0.11	*−0.95*	−0.05
calcium	*0.70*	0.64	−0.15
magnesium	*0.89*	0.31	−0.24
potassium	*0.88*	−0.25	−0.18
sodium	*0.74*	−0.48	−0.21
boron	*0.82*	−0.27	0.04

### 2.3. Data analysis

We simplified our data on soil fertility by using a principal components analysis to identify major trends in variation among the different elemental concentrations as well as conductivity and pH, and to extract a single variable that represented correlated variation in many of these variables and was interpretable as a measure of soil fertility. We then used a mixed-effects regression model to test for effects of digging on this measure of soil fertility, by contrasting measurements on soil samples from three digging conditions: soil taken from the foraging pits themselves, from spoil heaps beside foraging pits and from adjacent areas undisturbed by digging mammals. Because these samples were collected as triplets at each plot, we entered plot ID as a random effect to control for non-independence of soil samples among the three conditions. The model also included the effects of the three main habitat types to investigate the additive and interactive effects of habitat as well as digging treatment on soil fertility. Thus, the model took the form: Soil Fertility PC1 ∼ Habitat Type + Digging Treatment + Habitat Type × Digging Treatment + (1|Plot ID).

We used the same modelling approach to investigate the relationships of digging and habitat to soil hardness measured by penetrometer force and to soil moisture, except that for these variables we included number of days since most recent rainfall as an additional predictor covariate. This was necessary because while all samples were collected at the same time of year, they were collected on different days, and differences in time since rain could affect soil hardness and moisture content. Thus, these models took the form: Penetrometer Force or Soil Moisture Content ∼ Days Since Rainfall + Habitat Type + Digging Treatment + Habitat Type × Digging Treatment + (1|Plot ID). All models were built using a Bayesian framework in R [25] using the package MCMCglmm, with *p*-values profiled using Markov chain Monte Carlo (MCMC) simulations. Because of the non-independence of the soil samples in relation to digging condition, we additionally evaluated the amount of variation explained by plot ID in the model by dividing the variance explained by the random effect (plot ID) with the sum of the variance from the random and fixed effects.

## 3. Results

### 3.1. Physical impacts of diggings

**Figure 1.**
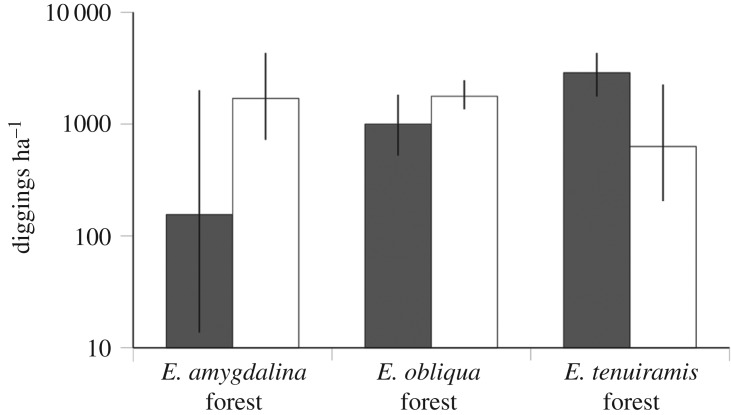
Densities of diggings by the eastern bettong (dark bars) and short-beaked echidna (white bars) in three major habitat types. Values are means ±95% confidence intervals.

The mean standing density of diggings from four surveys of 40 plots was 5812 (±909) diggings ha^−1^, ranging from 875 to 12 450 ha^−1^ at individual plots. Of all diggings, 41% were made by eastern bettongs, 42% by short-beaked echidnas, 3% by bandicoots (probably southern brown bandicoots, but diggings of the two species present in the area could not be distinguished) and 14% were unidentified. Density of eastern bettong diggings varied among habitat types, being generally low (but highly variable among plots) in *E. amygdalina* forest, and consistently high in the other two habitat types, especially *E. tenuiramis* forest. Density of echidna diggings was similar among the three major habitat types (figure 1).

The mean area disturbed by each digging was 0.19 (±0.02) m^2^, of which approximately 40% was the excavated pit and 60% the spoil heap. The average pit was roughly an elliptical cone or semi-ellipsoid with width 18.29 (±0.44) cm, length 15.45 (±0.37) cm, depth 14.40 (±0.50) cm and volume 0.0021 (±0.0007) m^3^. Diggings made by bettongs were generally larger than those of echidnas, disturbing nearly twice the surface area (on average 0.26 m^2^ compared to 0.14 m^2^) and with proportionally larger spoil heaps (63% of the disturbance compared to 55%) and pits (0.035 m^2^/0.022 m^2^ surface area, 0.0034 m^3^/0.0011 m^3^ volume, respectively). Bandicoot diggings were 0.00075 m^3^ and unknown diggings averaged 0.0016 m^3^.

On average, diggings for all species affected 11% (±3) of the forest floor, consisting of pits 4.4% (±1.2) and spoil heaps 6.6% (±1.8), and represented 12.2 (±4.06) m^3^ of excavated soil per hectare. Diggings disturbed 6.5% (±2.1) of the forest floor annually, displacing 7.1 (3.7–11.6) m^3^ ha^−1^ yr^−1^ of soil. New diggings were created at a rate of 2377 (±499) ha^−1^ yr^−1^, and diggings decayed to non-detectability at 3113 (±798) ha^−1^ yr^−1^, except that some old diggings were re-excavated (1018 ± 256 ha^−1^ yr^−1^). Excluding re-excavations, new digging disturbed between 3.2 and 6.0% (mean 4.5%) of the forest floor annually, excavating 5.0 (2.6–8.1) m^3^ ha^−1^ yr^−1^ of the previously undisturbed soil. Of 196 re-excavations identified to species, 131 were by echidnas and 65 by bettongs; 137 were originally dug by bettongs and 59 by echidnas. Re-excavated diggings had average length (21.27 cm), width (19.31 cm), depth (15.83 cm) and volume (0.0036 m^3^), close to the average size of bettong diggings, and the largest echidna diggings.

### 3.2. Effects of diggings on soil fertility, hardness and moisture

The first PC axis accounted for 46.98% of soil-composition data. Scores on PC1 were strongly correlated with conductivity and organic carbon concentration, as well as with concentrations of elements such as phosphorus, potassium, sulfur, copper, zinc, calcium, magnesium, sodium and boron, and were positively but less strongly correlated with nitrogen (table 1). We interpret this PC as representing general soil nutrient availability, and in subsequent analysis we use it to represent soil fertility.

**Figure 2.**
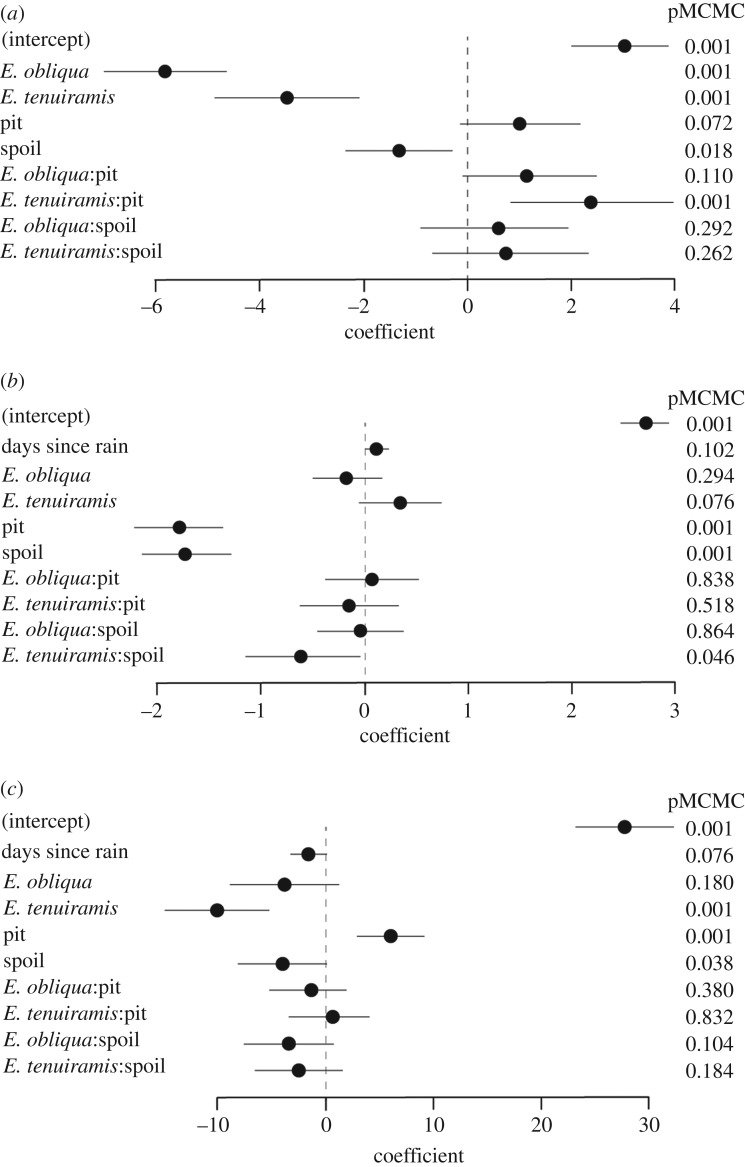
Coefficient sizes (±95% credible intervals) and associated *p*-values of the effects of habitat types, digging treatment, and the interaction between habitat type and digging treatment (that is, soil taken from pits dug by mammals, from spoil heaps created as a result of digging and from undisturbed ground), on (*a*) soil fertility (first principal component of soil composition), (*b*) penetrometer force (representing soil hardness) and (*c*) soil moisture content. Coefficient sizes and *p*-values are relative to *E. amygdalina* (for habitat) and undisturbed topsoil (for digging treatment).

Values of PC1 varied among habitats and digging treatments (figure 2*a*). *Eucalyptus tenuiramis* and *E. obliqua* forest had lower soil fertility than *E. amygdalina* forest. Fertility was higher in the soil samples taken from foraging pits than in the samples of undisturbed soil, and lower in the samples from spoil heaps, but these differences were affected by interactions with habitat. Fertility of soil from foraging pits was elevated with respect to undisturbed soil in the *E. obliqua* and *E. tenuiramis* forest habitats, which were both of generally low fertility, while the fertility of soil from spoil heaps in those habitats did not differ from that of undisturbed soil (figure 2*a*). Overall, plot ID explained 54.2% (CI 35.4–72.9) of variation in soil fertility.

Soil penetration resistance (representing hardness) was not significantly related to habitat or days since rainfall but was strongly affected by the digging treatment: soil from pits and spoil heaps was much less resistant than undisturbed soil (figure 2*b*). Plot ID explained a negligible amount of variation in penetration resistance (less than 0.01%). Days since rain tended to have a negative effect on soil moisture content (figure 2*c*), and soil moisture was also lower in *E. tenuiramis* than *E. amygdalina* forest. Soil moisture was strongly affected by digging treatment, being high in soil from foraging pits and low in soil from spoil heaps, relative to undug soil in all habitats. Overall, there was a strong effect of plot ID on soil moisture of 77.2% (CI 60.9–86.5).

## 4. Discussion

Digging by mammals had significant effects on soil. The digging of foraging pits, followed by the passive infilling of those pits with mixed soil and litter, created patches of loose and nutrient-rich soil that retained higher moisture content than surrounding soil. Spoil heaps thrown out of foraging pits formed patches of bare ground. Digging therefore created a fine-scaled patchwork of differing ground cover and edaphic conditions. Given the high rate at which new diggings were excavated and old diggings disappeared as a result of infilling, this patchwork was dynamic and produced a shifting pattern of fine-scaled disturbance across the forest floor. The effects of digging ameliorated other environmental stresses, because the localized increase in soil fertility due to digging was greatest in habitats of lower fertility and increases in soil moisture were greatest in habitats where soil moisture was otherwise low.

This was the first study of the effects of bioturbation by native mammals in a temperate dry sclerophyll forest environment. It was also the first such study in Tasmania, where all of the original native digging mammals are extant. The few measurements of diggings by medium-sized native mammals that have been made in other parts of Australia suggest that digging activity can be comparable to values recorded here and may generally be higher in woodland environments than in arid shrublands and grasslands. However, these high values are found only where populations of critical-weight-range marsupials are wholly or partially protected by control of invasive predators. In dry woodland in Western Australia, where red foxes are controlled by extensive poison baiting, a relict population of the critically endangered woylie *Bettongia penicillata* excavated 5000–16 000 diggings, and displaced an estimated 1.6–4.0 tonnes of soil, per ha per year [11]. These digging rates were approximately doubled in a small predator-free fenced reserve in Western Australia [16]. The combined efforts of reintroduced greater bilbies *Macrotis lagotis* and burrowing bettongs *Bettongia lesueur* in a large predator-free exclosure in an arid environment produced around 1100 diggings ha^−1^ representing 4.29 tonnes ha^−1^ of displaced soil [26]. In a semi-arid environment open to invasive predators, densities of diggings by echidnas varied from approximately 120 to 400 ha^−1^, depending on habitat [27], considerably less than the densities of digging by echidnas recorded in this study. Study of the activity of individual echidnas indicated that each animal can displace around 204 m^3^ of soil each year [28], suggesting that even at quite low densities, echidnas can have large impacts on soil structure.

The eastern bettong and short-beaked echidna were responsible for most of the digging activity in our study area. Digging by bettongs was variable in space, being greatest in drier forests with open heath understorey vegetation. This result is consistent with previous research that found positive relationships between density of bettong diggings and characteristics of dry heathy forest, especially extent of bare ground and stem densities of *Eucalyptus tenuiramis* and *Acacia dealbata* [29]. In wetter and denser forest types in Tasmania, the eastern bettong is replaced by the long-nosed potoroo. Although we did not measure the densities of diggings produced by long-nosed potoroos, our observations in other sites suggest that this species is responsible for levels of digging activity at least as high as those we measured for eastern bettongs. We suggest, therefore, that bioturbation by marsupials is a prominent characteristic of a wide range of Tasmanian environments. Digging by rat-kangaroos varies in time as well as in space. For example, digging rates increase soon after fire, apparently as animals exploit increased availability of their main food—sporocarps of hypogeous ectomycorrhizal fungi—in the aftermath of fire [21,30]. Also, populations of the eastern bettong in Tasmania evidently fluctuate in response to climate variation on timescales of several years (K. Proft 2018, personal communication). These variations are likely to cause changes in the scale of digging that could affect the temporal dynamics of other plant and animal communities, but our study was of insufficient duration to measure these changes.

Echidna digging activity was similar across different soil and vegetation types in our study area, suggesting that echidnas did not favour particular environments for foraging. This may be because their food (invertebrates, especially ants) is abundant and widespread in Tasmanian forests and woodlands. Elsewhere, variation in densities of diggings by echidnas has been linked only to the availability of shelters [31].

Our results support other studies that have found strong effects of digging by medium-sized Australian mammals on soil characteristics [32–34]. These effects consist, first, of direct physical displacement and loosening of soil. Digging of foraging pits also mixes soil from surface and sub-surface layers, and it incorporates litter into soil because some litter is buried under spoil heaps [35,36]. Loosening of soil by bettongs and other mammals allows higher water infiltration [10,37]; also, the foraging pits themselves capture water that would otherwise flow across the undisturbed soil surface and be partially lost from the local habitat.

Foraging pits act as traps that accumulate mobile debris as well as moisture. Our data suggest that pits were quite efficient traps, given that they were typically infilled over periods of one or two years. We did not study the process of accumulation of material in foraging pits, but two distinct mechanisms appear to be involved. First, material that is deposited directly into pits as part of the general fall of leaves, twigs and bark from the shrub and tree company is protected from wind and water and so is unlikely to be secondarily displaced. Second, material that is moved laterally across the surrounding soil surface by wind or water may come to rest in pits, preventing further displacement; such material would include fine soil particles and microfauna as well as litter. More subtly, spoil heaps probably also impede lateral movement of these soil and organic fractions, which accumulate in drifts where their directional movement encounters spoil heaps. The effect of these processes is to cause a redistribution of such material, and to concentrate it in microsites disturbed by mammal diggings, especially in the pits themselves. Perhaps more importantly, diggings probably reduce the total loss of such material from areas—on sloping ground, for example—that would otherwise be susceptible to mass erosion of mobile soil and organic fractions by wind and water. Consequently, mammalian diggings not only cause fine-scaled redistribution and concentration of soil particles and organic matter, but may also provide insurance against the loss of such material at broader scales.

A general result of the processes described above is that soil nutrients are retained and concentrated in micro-sites disturbed by mammal diggings. Soil that formed in foraging pits had higher nutrient availability than undisturbed soil. The difference was large, of a magnitude that approached differences in soil fertility across major habitat contrasts in our study area. Other studies have also reported elevated soil nutrient status in foraging pits created by Australian mammals [32–34,38,39]. These differences have significant effects on plant growth, leading to higher growth rates of grasses, shrubs and trees in soil taken from foraging pits than from surrounding undisturbed soil [35,38,40].

We found that existing diggings were often re-excavated. This could be because foraging mammals dig in the sites that are most productive for the foods they seek (in this case, those foods were predominantly hypogeous fungi for eastern bettongs, and invertebrates for short-beaked echidnas), and they are therefore more likely to re-dig in the same sites. However, the high frequency of cases of previous diggings being precisely re-dug suggests another possibility: that initial disturbance by mammals promotes replenishment of the same resources, so that sites become more productive once they have been disturbed, and the animals responsible for the initial disturbance preferentially return to harvest those replenishing resources. If this speculation is correct, re-excavation of diggings could represent a form of niche construction by digging mammals, in which their activities cause a shift in resource productivity and hence an increase in carrying capacity for those same animals. Positive resource utilization–production feedback is a characteristic of some other ecosystem engineers, such as the North American beaver *Castor canadensis* [41]. An alternative explanation for re-excavation of old pits is that the looseness of soil in such pits makes re-digging easier, shifting the cost–benefit ratio for digging in favour of previously dug rather than novel sites.

The eastern bettong is extinct on mainland Australia, and persists only in Tasmania, except for a recently established mainland population in a fenced reserve [42]. The two species of bandicoots that persist in Tasmania are also rare on mainland Australia, while the long-nosed potoroo, still abundant in Tasmania, has declined in southeastern mainland Australia [43]. More generally, medium-sized mammals that dig for their food in the ecosystems of mainland Australia have declined dramatically since European settlement [17,43]. Our data support the hypothesis that the loss of digging species has changed soil characteristics, reduced soil fertility and degraded ecosystem functioning over large areas of Australia [16,44]. For these reasons, reintroduction of digging mammals such as the eastern bettong that have become extinct from large parts of their original ranges should be a crucial element of ecological management and restoration of ecosystems on mainland Australia.

## Supplementary Material

Soil data all sites

## Permission to carry out fieldwork

Permits to conduct work in the study area and remove soil samples were provided by the Tasmanian Department of Primary Industries, Parks, Wildlife and Environment.

## Data accessibility

Data are available as electronic supplementary material.
